# Accelerated Apoptosis of Neutrophils in Familial Mediterranean Fever

**DOI:** 10.3389/fimmu.2015.00239

**Published:** 2015-05-18

**Authors:** Gayane Manukyan, Rustam Aminov, Gagik Hakobyan, Tigran Davtyan

**Affiliations:** ^1^Group of Molecular and Cellular Immunology, Institute of Molecular Biology, National Academy of Sciences, Yerevan, Armenia; ^2^National Veterinary Institute, Technical University of Denmark, Copenhagen, Denmark; ^3^Department of Internal Medicine, Yerevan State Medical University, Yerevan, Armenia; ^4^Analytical Laboratory Branch, Scientific Centre of Drug and Medical Technology Expertise JSC, Yerevan, Armenia

**Keywords:** familial Mediterranean fever, autoinflammation, apoptosis, neutrophils, whole blood

## Abstract

The causative mutations for familial Mediterranean fever (FMF) are located in the *MEFV* gene, which encodes pyrin. Pyrin modulates the susceptibility to apoptosis via its PYD domain, but how the mutated versions of pyrin affect apoptotic processes are poorly understood. Spontaneous and induced rates of systemic neutrophil apoptosis as well as the levels of proteins involved in apoptosis were investigated *ex vivo* in patients with FMF using flow cytometry and RT-qPCR. The freshly collected neutrophils from the patients in FMF remission displayed a significantly larger number of cells spontaneously entering apoptosis compared to control (6.27 ± 2.14 vs. 1.69 ± 0.18%). This elevated ratio was retained after 24 h incubation of neutrophils in the growth medium (32.4 ± 7.41 vs. 7.65 ± 1.32%). Correspondingly, the mRNA level for caspase-3 was also significantly increased under these conditions. In response to the inducing agents, the neutrophils from FMF patients also displayed significantly elevated apoptotic rates compared to control. The elevated rates, however, can be largely explained by the higher basal ratio of apoptotic cells in the former group. Monitoring of several proteins involved in apoptosis has not revealed any conventional mechanisms contributing to the enhanced apoptotic rate of neutrophils in FMF. Although the exact molecular mechanisms of accelerated neutrophil apoptosis in FMF remain unknown, it may provide a protection against excessive inflammation and tissue damage due to a massive infiltration of neutrophils in the acute period of the disease.

## Introduction

Familial Mediterranean fever [FMF, MIM249100] is an autoinflammatory syndrome characterized by the recurrent episodes of fever and polyserositis. The underlying cause of the disease is mutations in the *MEFV* gene, which encodes the protein called pyrin or marenostrin (1, 2). The protein is primarily expressed in neutrophils, eosinophils, and cytokine-activated monocytes (3), and it is thought it regulates the activation of caspase-1 and IL-1β (4–6). Via the PYD domain, pyrin interacts with ASC (apoptosis-associated speck-like protein with a caspase recruitment domain), which overexpression is closely associated with inflammation and apoptosis (7, 8). ASC is a central adaptor protein of the inflammasome, which mediates interaction of the pathogen recognition receptors and caspase 1 (9). Despite the controversies regarding the exact mechanisms by which the mutated pyrin versions cause the episodes of sterile inflammation, it is believed that pyrin influences formation of yet unknown inflammasome, a multiprotein complex that mediates activation of caspase-1 and subsequent secretion of IL-1β. In a number of studies, the NALP3 inflammasome complex has been implicated in the pathogenesis of FMF (6, 10); whereas in other works, it has been associated with its own, pyrin inflammasome (11).

The hallmark of FMF is the self-limiting periods of intense inflammation, which is mainly mediated by a massive influx of polymorphonuclear neutrophils (PMNs) into the inflamed sites (12). Remission between the episodes of acute inflammation is also characterized the enhanced pro-inflammatory activity of circulating PMNs, thus suggesting a subclinical inflammation in FMF (13). Although a chronic pro-inflammatory state of neutrophils could be detrimental to the host, it has been suggested that, in the past, this FMF feature had provided an increased resistance to an as yet unidentified infectious agent and thus the carriers of *MEFV* mutations had better survival rates (14, 15). The state of activated PMNs, however, could be harmful to tissues, and the timely elimination of them via apoptosis is an important biological function for the successful resolution of inflammation and prevention of tissue damage (16). The apoptotic activity of neutrophils is constitutive (17), and it can be influenced by a number of extracellular factors such as host-derived cytokines, extracellular nucleotides, and pathogen-derived ligands (18–20), which can accelerate or suppress the life span of neutrophils (21). The intrinsic activation of the apoptotic pathway in neutrophils may also happen in response to cellular stress and damage, and it is tightly controlled by the balance of pro- and anti-apoptotic proteins of the Bcl-2 family (22).

Despite the importance of apoptosis in the down-regulation and eventual resolution of the inflammatory response, there is limited information available regarding the apoptotic activity of neutrophils in FMF, and the results are contradictory. In particular, some of them suggested the accelerated rate of spontaneous apoptosis during the acute flares (23), while others reported no changes in the rate of PMNs apoptosis (24). The main aim of our work was to investigate whether the rates of spontaneous apoptosis of neutrophils in FMF are different compared to the norm. We also compared the level of apoptosis induction in neutrophils from the FMF and control cohorts in response to the external signals, such as tumor necrosis factor alpha (TNFα), lipopolysaccharide (LPS), adenosine triphosphate (ATP), muramyl dipeptide (MDP), synthetic bacterial lipopeptide (Pam3CSK4), and colchicine. We also monitored the expression of proteins involved in apoptosis such as anti-apoptotic MCL-1, heat shock protein 70 (Hsp70), tumor suppressor protein p53, and caspase-3 in neutrophils from the FMF and control subjects.

## Materials and Methods

### Patients

Two groups of volunteers were recruited for the study. The first group included 16 FMF patients during the disease remission (12 males, 4 females; age 14–41 years, mean age 24.2 years). The clinical diagnosis of FMF was based on Tel-Hashomer criteria (25). Genetic confirmation for the *MEFV* mutation carrier status was performed by the Centre of Medical Genetics (Yerevan, Armenia). Determination of the disease stage was based on clinical and laboratory findings (fever, FMF-related symptoms, such as abdominal and thoracic pain, arthritis, high levels of C-reactive protein, white blood cell counts, and erythrocyte sedimentation rate). The remission phase was defined as being free of these symptoms for at least 2 weeks. Peripheral blood samples from patients with FMF were obtained during the visits to the Department of Gastroenterology and Familial Mediterranean Fever, Medical Centre Armenia (Yerevan, Armenia). All FMF patients in the study did not receive any colchicine treatment before. The control group consisted of 11 sex- and age-matched healthy individuals (7 males, 4 females; age 22–39 years, mean age 27.3 years) with no personal or family history of FMF. Peripheral blood of healthy controls was provided by the blood bank of the Viola company (Yerevan, Armenia). Written informed consents were obtained from all study participants. The study was approved by the Ethical Committee of the Institute of Molecular Biology of the NAS RA (IRB IORG0003427).

### Sampling and whole blood cell culture

The rate of apoptosis and expression of MCL-1, Hsp70, and p53 were determined in neutrophils from the freshly collected venous blood samples of FMF and control subjects. The blood samples were collected in sterile EDTA-coated tubes and processed within 2 h after the blood collection. The whole blood samples (100 μl) were incubated at 37°C in the RPMI-1640 medium supplemented with 10% fetal calf serum, 2 mmol/l l-glutamine, 1 mM sodium pyruvate, 5 mM HEPES, 100 units of penicillin, and 100 μg of streptomycin. The effect of the following agents was tested during the 3 and 24 h incubations: TNFα and LPS (at final concentration of 10 ng/ml), ATP-γ-S (100 μM/ml), MDP and Pam_3_CSK4 (2.5 μg/ml), and colchicine (2 μg/ml). After the incubation, blood cells were drawn from the culture, washed with PBS, and the contaminating erythrocytes were removed by brief hypotonic lysis.

### Measurement of PMN apoptosis

The apoptotic index of neutrophils was quantified by staining the white blood cells with biotin-conjugated Annexin V (BD Bioscience). After the incubation with the saturated concentration of biotin-conjugated Annexin V, the cell suspension was further incubated with PE-conjugated Streptavidin (BD Bioscience). The positively stained apoptotic cells were counted, and the apoptotic index was calculated as the percentage of apoptotic cells within the total number of cells.

### Measurement of intracellular protein expression

The level of intracellular proteins p53 and Hsp70 in neutrophils was measured in the whole blood cells by immunofluorescent antibody staining and analyzed by quantitative flow cytometry. Briefly, the cell suspension was fixed in the solution with formaldehyde (final concentration 2%) and stained with the FITC-conjugated mouse monoclonal [C92F3A-5] anti-human Hsp70 and PE-conjugated mouse [DO-1] anti-human p53 (Abcam, UK) in the permeabilization solution (0.1%, eBioscience). The isotype-matched IgG2a (FITC) and IgG1 (PE) antibodies (eBioscience) were used throughout the experiments as negative controls for Hsp70 and p53, respectively. The dual-fluorochrome spectral overlap was compensated by using the cells separately stained with the FITC- or PE-labeled antibodies. Approximately, 10,000 events were collected per sample. Forward scatter and side scatter were used to identify the PMN population and to separate other cells (monocytes and lymphocytes) and debris. Data were analyzed using the CellQuest software (BD Biosciences), and the mean fluorescence intensity (MFI) as well as the percentage of apoptotic cells were used to quantify the responses to the agents listed above.

The level of intracellular MCL-1 in neutrophils was stained with mouse monoclonal [8C6D4B1] MCL-1 primary antibody (Abcam, UK). The secondary antibody treatment was with anti-mouse IgG biotin (eBioscience), which was followed by staining with FITC-conjugated Streptavidin (eBioscience, UK).

### PMNs culture

In brief, venous blood (12 ml) was collected into the sterile vacutainer tubes containing EDTA (Vacutest Kima s.r.l., Arzergrande, Italy). Peripheral blood neutrophils were isolated by density centrifugation using Ficoll-Hypaque gradient. Erythrocytes contaminating the granulocyte fraction were removed by brief hypotonic lysis. The purity of granulocytes, as determined by counting the cytospin preparations stained with May-Grundwald Giemsa, was consistently >95%. The viability of neutrophils, as assessed by trypan blue exclusion, was >95%, immediately after the purification.

The purified neutrophils (1 × 10^6^ cells/ml) were cultured in polypropylene tubes in the RPMI medium 1640 supplemented with 10% fetal calf serum and 2 mmol/L of l-glutamine in a total volume of 1 ml for 24 h at 37°C. After the growth, neutrophils were washed from the cultivation media with cold PBS.

### RNA extraction and RT-qPCR

Total RNA isolation, cDNA synthesis and qPCR were performed as described previously (13). qPCR assay was conducted with the TaqMan assay primers (CASP3: Hs.PT.47.2840197; Integrated DNA Technologies) and with in-house developed primers and probes with the following oligonucleotide sequences: RPL32 (forward) – 5′-GAA GTT CCT GGT CCA CAA CG-3′, RPL32 (reverse) – 5′-GCG ATC TCG GCA CAG TAA G-3′, and RPL32 LNA probe #17. The RPL32 gene expression was used for the normalization of mRNA concentration in neutrophils. The human universal reference RNA (Stratagene, La Jolla, CA, USA) was used as a calibrator at the concentration of 1.25 ng RNA/reaction in quadruplicates (26). The relative expression level was calculated using the second derivative method (Rotor Gene software 6.1.71, Corbett Research).

### Statistical analysis

Statistical analyses were carried out using the Statsoft Statistica package (http://www.statsoft.com). For continuous variables, groups were compared using a two-sample *t*-test or a Mann–Whitney *U* test if data were not normally distributed. The percentage of the apoptotic cells is given as mean ± SEM. *p*-values <0.05 were considered as significant.

## Results

### Apoptosis rates in PMN cells

**Figure 1:**
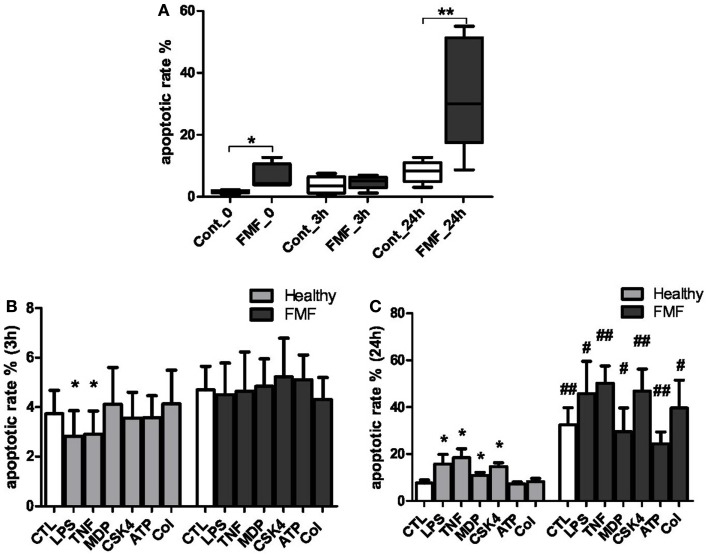
**(A)** Spontaneous apoptotic rate of neutrophils in the whole blood of FMF patients (FMF; *n* = 8) and healthy subjects (Cont; *n* = 8) in culture (media): 0 – basal apoptotic rate; 3 h – spontaneous apoptotic rate of the cells cultured in media (untreated) for 3 h; 24 h – spontaneous apoptotic rate of the cells cultured in media (untreated) for 24 h. Data are represented as the % annexin V+ cells (mean ± SEM). *p < 0.05, **p < 0.01 – significant value. **(B,C)** Effects following inducers: LPS, TNF-α, MDP, Pam_3_CSK4 (CSK4), ATP, colchicine (Col) on the apoptotic rate of circulating neutrophils in the blood of patients with FMF and healthy subjects (CTL) cultured for 3 **(B)** and 24 h **(C)**. *p < 0.05 vs. untreated cells; ^#^*p* < 0.05, ^##^*p* < 0.01 vs. healthy PMNs. Data are represented as the % annexin-V+ cells (mean ± SEM).

To evaluate the impact of FMF on the apoptotic processes in systemic PMNs, we measured the spontaneous rates of apoptosis in the cells from FMF patients compared to healthy subjects at 0, 3, and 24 h (Figure 1A). Also, the corresponding rates were estimated for the cells treated by LPS, TNFα, MDP, CSK4, ATP, and colchicine for 3 and 24 h (Figure 1B).

### Spontaneous apoptotic rates

The spontaneous apoptotic rates of systemic neutrophils from FMF patients were found to be significantly higher compared to the cells from healthy subjects. The basal (0 h) level of apoptotic PMNs in the whole blood of FMF patients exceeded the corresponding values of the healthy subjects by 3.7-fold (6.27 ± 2.14 and 1.69 ± 0.18%, respectively; *p* < 0.05) (Figure 1A). No significant differences in the apoptotic rates of neutrophils from both sources were detected following a 3 h incubation period. The incubation resulted in a minor increase of the apoptotic rate in control PMNs (from 1.69 to 3.72%, *p* = 0.11), while the opposite trend, with a slight decrease (from 11.5 to 4.7%, *p* = 0.27), was observed for the FMF neutrophils. Following the 24 h incubation, however, the proportion of apoptotic PMN cells in the blood of FMF patients was more than fourfold higher compared to the control group (32.4 ± 7.41 and 7.65 ± 1.32%, respectively; *p* < 0.01) (Figure 1A).

### Induced apoptotic rates

The effect of various ligands, which may differentially affect the rates of apoptosis in systemic neutrophils from the diseased and healthy subjects, was evaluated with the cells incubated in the presence of LPS, TNFα, MDP, CSK4, ATP, and colchicine. About 3 h exposure to LPS caused a small but significant delay of apoptosis in neutrophils from the healthy subjects (3.72 ± 0.94 and 2.81 ± 1.04% for the non-exposed and LPS-exposed neutrophils, respectively; *p* = 0.05) (Figure 1B). The short exposure of neutrophils from the healthy subjects to TNFα also resulted in a delayed apoptosis (3.72 ± 0.94 and 2.9 ± 0.94% for the non-exposed and TNFα-exposed neutrophils, respectively; *p* = 0.05) (Figure 1B). The 3 h exposure of neutrophils from healthy controls to other ligands (MDP, CSK4, ATP, and colchicine) had no discernible impact on the percentage of apoptotic cells. No significant differences in the apoptotic rates of neutrophils exposed to 3 h incubation with the ligands were found between the diseased and healthy subjects (Figure 1B).

A more prolonged 24 h incubation of blood from the healthy subjects resulted in a significant increase of the apoptotic rate in neutrophils in response to certain ligands. These were: LPS (15.76 ± 4.12 and 7.65 ± 1.32%; *p* < 0.05), TNFα (18.44 ± 3.81 and 7.65 ± 1.32%; *p* < 0.05), MDP (10.86 ± 1.29 and 7.65 ± 1.32%; *p* < 0.05), and CSK4 (14.61 ± 1.66 and 7.65 ± 1.32%; *p* < 0.05). The incubation with ATP and colchicine had no significant effect on the rate of apoptosis in this cohort (Figure 1B).

On the contrary, the PMN cells from the FMF patients were much less responsive to the 24 h incubation with the ligands in terms of the apoptotic rate (Figure 1C). When compared to the control group, however, the apoptotic rates were significantly elevated in response to all inducers used. Specifically, PMNs from the FMF patients displayed higher apoptosis rates compared to the control subjects in response to LPS (45.75 ± 13.8 vs. 15.76 ± 4.12%; *p* < 0.05), TNFα (50.17 ± 7.41 vs. 18.44 ± 3.81%; *p* < 0.01, respectively), MDP (29.5 ± 10.21 vs. 10.86 ± 1.29%; *p* = 0.05), CSK4 (46.77 ± 9.49 vs. 14.61 ± 1.66%; *p* < 0.01), ATP (24.38 ± 5.04 vs. 7.25 ± 0.85%; *p* < 0.01), and colchicine (39.62 ± 11.86 vs. 8.22 ± 1.41%; *p* < 0.05). Need to note, however, that the basal spontaneous rate of apoptosis during the 24 h incubation of PMNs from the FMF patients was already significantly higher compared to the control samples in the absence of any ligand (Figures 1A,C). Thus, the differences described above can be largely explained by the high basal spontaneous rate of apoptosis in PMNs from the FMF patients compared to the control subjects.

### Caspase-3 mRNA level

**Figure 2:**
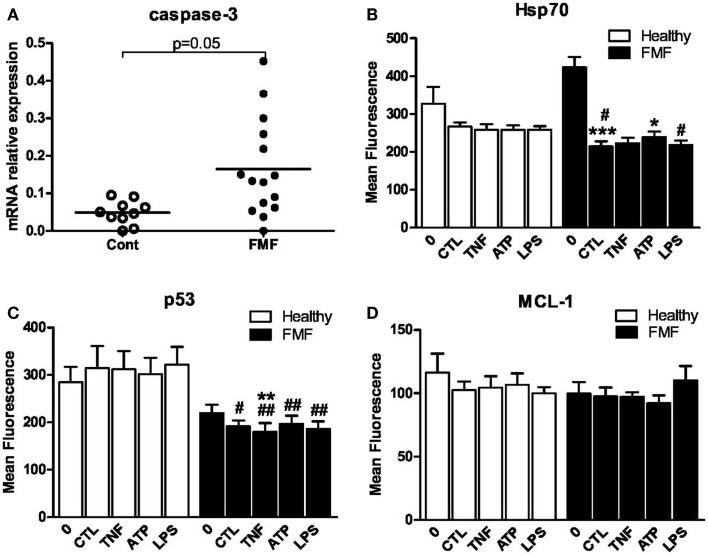
**(A)** Scatterplot showing caspase-3mRNA levels in isolated and cultured (24 h) in RPMI-1640 medium PMNs from FMF patients (FMF; *n* = 16) and healthy subjects (Cont; *n* = 11) as assessed by RT-qPCR. The expression of caspase-3 was normalized to that of RPL32. Horizontal bars represent the mean values for each group. **(B–D)** Effect of the 3 h exposure of neutrophils from FMF and control to TNFα (10 ng/ml), ATP (100 μM), and LPS (10 ng/ml) on intracellular expression of Hsp70 **(B)**, p53 **(C)**, and MCL-1 **(D)**. CTL – control medium. Values are the mean ± SEM of fluorescence intensity. *p < 0.05 vs. untreated cells; ^#^*p* < 0.05 vs. healthy PMNs; ^#^*p* < 0.05, ^##^*p* < 0.01 vs. healthy PMNs.

One of the key mediators of apoptosis is caspase-3, and we therefore assessed its expression in the freshly isolated neutrophils as well as in neutrophils cultured in the media by RT-qPCR. The baseline level of the caspase-3 transcript in neutrophils was not different between the FMF and control groups. The transcript level in the former group, however, tended to be significantly higher compared to the latter after the 24 h cultivation of neutrophils only with media (0.160 ± 0.037 vs. 0.052 ± 0.012; *p* = 0.05) (Figure 2A).

### Baseline and induced levels of Hsp70, p53, and MCL-1 in neutrophils

The level of proteins involved in regulation of apoptotic events such as Hsp70, p53, and MCL-1 was assessed in neutrophils from both cohorts using the flow cytometry technique. The effect of TNF-α, LPS, and ATP on the level of these proteins was measured following 3 h incubation with these agents. The levels of all three proteins in neutrophils from the control subjects were uniform and were not affected by the exposure to TNFα, LPS, and ATP (Figures 2B–D). The baseline levels of Hsp70, p53, and MCL-1 were not different between the two investigated groups. However, a slight increase in Hsp70 and decrease in p53 expression in neutrophils from the FMF patients tended to be significant. The MFI corresponding to the Hsp70 level in PMNs from the FMF patients was increased compared to the control subjects (422.6 ± 27.7 #MFI vs. 326.9 ± 44.7; *p* = 0.07) (Figure 2B). On the contrary, the level of p53 in PMNs was slightly decreased in the diseased group compared to control (219.4 ± 18.3 vs. 284.4 ± 32.9; *p* = 0.08) (Figure 2C).

Cultivation of PMNs from the FMF patients in the media without the ligand addition for 3 h led to the drastic twofold decrease in the Hsp70 level (422.6 ± 27.7 and 214.5 ± 13.7, respectively; *p* < 0.001) (Figure 2B). Despite the initial higher level of Hsp70 in neutrophils from the FMF patients, the identical incubation conditions led to a more pronounced and significant fall of Hsp70 in the diseased group compared to control (214.5 ± 13.7 and 266.9 ± 11.1, respectively; *p* < 0.05) (Figure 2B). ATP exposure significantly increased the Hsp70 levels only in the FMF patient-derived cells (214.5 ± 13.7 and 238.5 ± 15.5, correspondingly; *p* < 0.05). LPS treatment resulted in a significant decrease of Hsp70 in FMF samples compared to control (218.7 ± 11.4 and 257.9 ± 10.1, respectively; *p* < 0.05). In general, despite the higher initial level of anti-apoptotic Hsp70 in neutrophils from FMF patients, they undergo accelerated apoptosis under the *in vitro* cultivation and ligand exposure conditions.

The level of p53, which controls p53-dependent apoptosis, was significantly lower in the PMN cells from FMF patients compared to healthy controls (191.5 ± 12.4 and 314.4 ± 47.1, respectively; *p* < 0.05) (Figure 2C). TNFα treatment resulted in a slight but significant decrease in p53 expression in the FMF neutrophils (191.5 ± 12.4 and 180.3 ± 18.2, respectively; *p* < 0.01). Decreased p53 levels were observed in FMF neutrophils compared to that of the healthy after the exposure to all inducers studied: TNFα (180.3 ± 18.2 vs. 312.2 ± 38.1; *p* < 0.01), ATP (196.8 ± 17.2 vs. 301.9.8 ± 34.6; *p* < 0.01), and LPS (186.3 ± 15.7 vs. 321.8 ± 37.6; *p* < 0.01). In general, and similarly to Hsp70, the level of p53 is suppressed under the *in vitro* cultivation and ligand exposure conditions. Thus, it is highly unlikely that the apoptotic pathway in neutrophils from FMF patients is p53-dependent.

There were no significant changes in the anti-apoptotic MCL-1 levels in the neutrophils from the both groups, irrespectively, whether or not these cells were subjected to the ligand treatment during the *ex vivo* incubation in the RPMI-1640 media (Figure 2D).

## Discussion

Here, we compared the rate of apoptosis among the peripheral neutrophils obtained from the FMF patients in remission and from the healthy control subjects. Our main finding is that the *ex vivo* spontaneous apoptotic rate of neutrophils from the former group is significantly higher compared to control, immediately after the isolation and after 24 h incubation in RPMI-1640 media. This observation was confirmed with two types of the 24 h *ex vivo* incubation, one using the whole blood samples and another – with the purified fraction of PMNs. In accordance with this, the level of mRNA for caspase-3, which is one of the main executioner caspases, was significantly higher in the PMNs from the FMF patients compared to control.

The acute inflammatory attacks in FMF are characterized by a massive influx of neutrophils into the affected sites, but these are usually self-limiting and do not cause tissue damage. Earlier studies reported the accelerated rate of apoptosis among the neutrophils during the disease attack, but no significant changes in the apoptotic rates in the remission period (23). Generally, shorter incubation times in the cited work may explain the discrepancy between our results. We also detected no difference in the apoptotic rates when the incubation time was reduced to 3 h.

Need to note here that even during the FMF remission periods, the peripheral PMNs remain activated (13). This may contribute to the subclinical inflammation in remission (27). The prolonged activation stage of PMNs, however, is not due to the delayed apoptosis. On the contrary, as we demonstrated here, the PMNs are characterized by the accelerated apoptosis.

How the mutated version of pyrin may contribute to the accelerated apoptotic rate of neutrophils? Pyrin possesses several functions and, depending on the internal and external cues, it may be involved in the pro- and anti-inflammotary activities as well as in apoptotic processes. The nature of the pyrin interaction with ASC has been investigated in a number of studies (8, 28, 29). There is a body of evidence for the pro-apoptotic effects of ASC (8, 28), as well as for the effect of pyrin as a modulator of the ASC-induced apoptotic process (8). Remarkably, ASC mRNA expression is significantly increased in the *MEFV* mutation-positive group compared to the mutation-negative group (30).

The multifunctional roles of pyrin and ASC, however, make it difficult to come up with a simple one-factor mechanistic model. In particular, pyrin can also interact with caspase-1 (4), suggesting the possibility for modulating yet another apoptotic pathway leading to the caspase-1-dependent cell death (31). A recently proposed paradigm has suggested that pyrin may have yet another function, serving as a pattern recognition receptor in the pyrin inflammasome (11). In this paradigm, pyrin is a specific immune sensor for pathogen modification and inactivation of Rho GTPases. In response to these cues, pyrin mediates the activation of caspase-1 inflammasome. Because of the broad variety of *MEFV* mutations, the protein encoded may have differential spatial conformations. This may result in different protein–protein interaction dynamic, thus leading to the extremely large variations in the apoptotic cell ratio in this group compared to the more tightly distributed range of apoptotic cells in the healthy neutrophils (Figure 1A).

We proposed earlier that PMNs in FMF are characterized by a heightened sensitivity toward stress/host-derived ligands (13), which could be a result of lower threshold concentrations needed for the activation of NALP3/pyrin inflammasomes. Continuous activation of the inflammasome in FMF, presumably by even normal physiological concentrations of ligands, can contribute to the enhanced ASC apoptotic signaling. Thus, pyrin-mediated modulation of ASC, which itself possesses a dual role in inflammation and apoptosis, may lead to the pro-inflammatory and pro-apoptotic pathways, which appear to be not mutually exclusive. Moreover, the apoptosome and the NLRP3 inflammasome share common activating factors such as the oxidized mitochondrial DNA (32). The main source of the oxidized mitochondrial DNA is the intracellular reactive oxygen species (ROS) that are produced by the activated neutrophils. ROS also participates in the initiation of apoptosis in neutrophils leading to the lysosomal damage and release of cathepsins, which, in turn, trigger the release of cytochrome C from mitochondria (33). It has been shown earlier that the neutrophils in FMF produce high levels of endogenous ROS (34).

The accelerated apoptosis rate of neutrophils from FMF patients during the 24 h *ex vivo* incubation in culture media is also supported by the elevated level of mRNA for caspase-3. The elevated mRNA expression of one of the main executioner caspases is consistent with the mechanism of the altered apoptosis in FMF neutrophils proposed above. We also demonstrated here that the FMF neutrophils cultivated in the presence of various signaling compounds respond differently to them in terms of the apoptotic rates than the neutrophils from the healthy subjects. PMNs from FMF patients are significantly less responsive to the external stimuli applied compared to the control cells. The higher percentage of apoptotic cells seen in the former group is simply the reflection of the higher spontaneous apoptotic rate.

It remains unclear, however, what are the endogenous stimuli that spontaneously trigger the neutrophils harboring the mutated version of pyrin into the pro-apoptotic pathway. They must be already present at point 0 conferring a 3.7-fold increase in the apoptotic rate of these susceptible neutrophils compared to the normal (Figure 1A). The same proportionally elevated level of apoptotic PMN cells in this group remains also after 24 h incubation in the growth media, suggesting that this is a self-sustainable process driven mainly by the internal factors. The nature of these endogenous factors remains elusive and further research is necessary to identify them.

We also investigated a possible involvement of several key apoptotic pathways in the accelerated apoptosis of PMNs with the mutated version of pyrin. We report, for the first time, that this apoptotic process does not involve the pathways associated with p53, Hsp70, and MCL-1. There was no correlation between the expression of these proteins and the accelerated apoptosis rate characteristic for the PMNs carrying the mutated pyrins.

## Conclusion

At the first glance, the accelerated apoptosis of neutrophils in FMF seems rather contradictory to the conventional view that the cells in the pre-activated state have the extended lifespan. There is a body of evidence that the *in vitro* induction of apoptosis in non-infectious inflammation models enhances the resolution of inflammation (35). We hypothesize here that the *MEFV* mutations have been selected not only for conferring the heightened sensitivity that allows triggering the immediate and non-specific innate inflammatory responses against the invading pathogens. The multi-functional pyrin, which is also involved in the apoptotic processes, may have been a good source for the selection of pleiotropic mutations that are simultaneously involved in the rapid onset of inflammation in response to infection as well as in rapid resolution of inflammation. Thus, the accelerated intrinsic apoptotic rates of neutrophils in FMF may have a protective effect in preventing tissue damage due to excessive inflammation.

## Conflict of Interest Statement

The authors declare that the research was conducted in the absence of any commercial or financial relationships that could be construed as a potential conflict of interest.
